# Polymyalgia Rheumatica Presenting as Fever of Unknown Origin

**DOI:** 10.31662/jmaj.2024-0196

**Published:** 2025-04-04

**Authors:** Hiroshi Shiba, Hiroki Ozawa, Masato Okada

**Affiliations:** 1 Immuno-Rheumatology Center, St Luke’s International Hospital, Tokyo, Japan; 2 Department of Rheumatology, Suwa Central Hospital, Nagano, Japan

**Keywords:** polymyalgia rheumatica, fever of unknown origin, PET/CT

A 76-year-old woman presented with persistent low-grade fever. She was initially suspected of blood culture-negative infective endocarditis. She underwent an aortic valve replacement and antibiotic treatment. The resected mass was, however, non-infective Lambl’s excrescences. ^18^-fluorine-fluorodeoxyglucose positron emission tomography/computed tomography (^18^F-FDG-PET/CT) was performed to detect foci of infection. Instead, it revealed FDG uptake, including around the shoulders and hips, in the interspinous regions and adjacent to the ischial tuberosities (Figure 1). She was referred to the rheumatology department. Focused history-taking revealed bilateral thigh pain with morning stiffness. Physical examination showed edematous extremities and limited range of motion in the wrists and ankles. Ultrasonography identified bilateral biceps tenosynovitis and distal synovitis. Rheumatoid factor and anti-cyclic citrullinated peptide antibodies were negative. She was diagnosed with polymyalgia rheumatica (PMR). Lambl’s excrescences were considered a coincidental finding unrelated to PMR.

**Figure 1.**
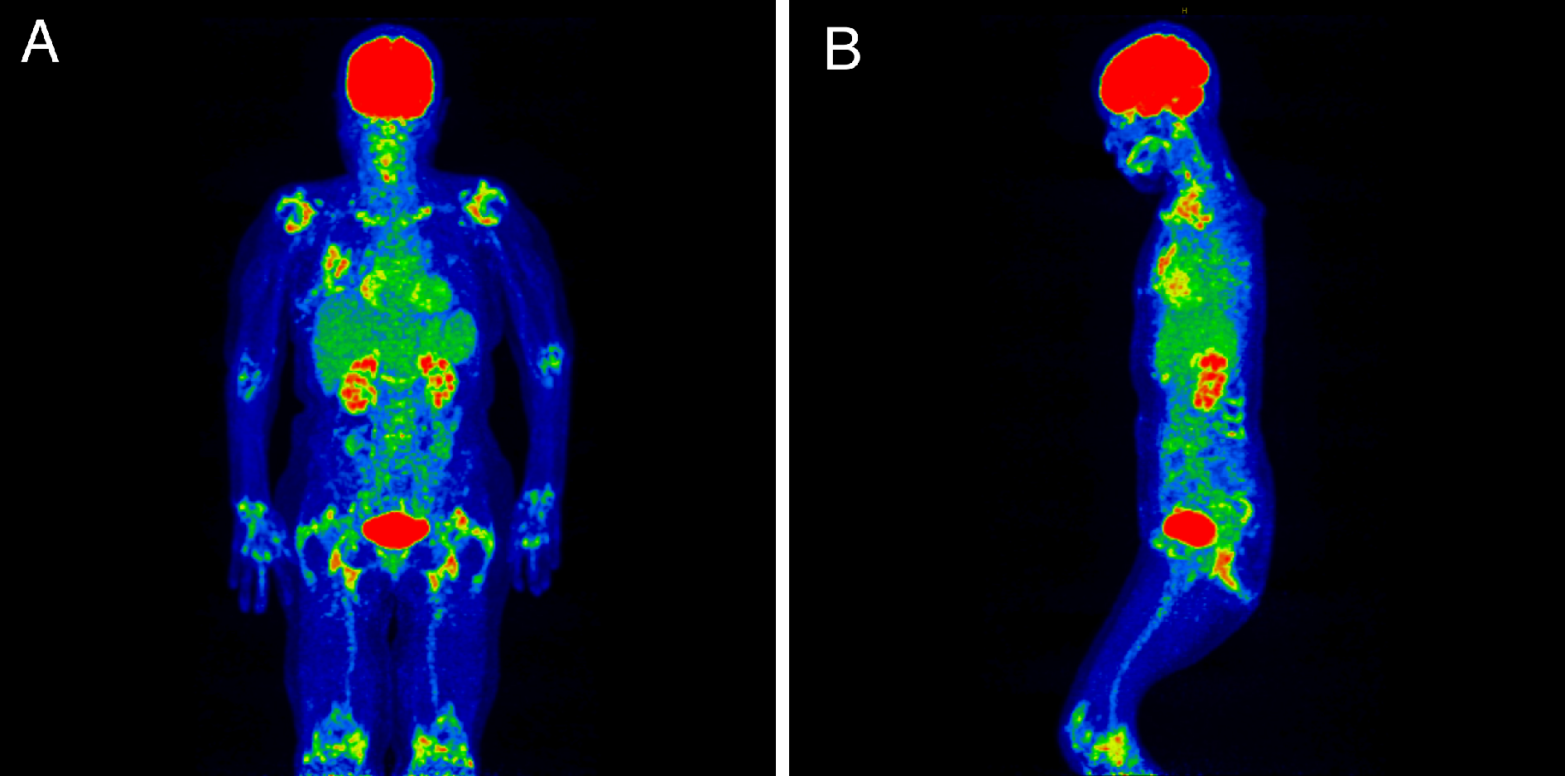
(A) 18-fluorine-fluorodeoxyglucose positron emission tomography/computed tomography (^18^F-FDG-PET/CT) of a 76-year-old woman, showing FDG uptake in bilateral shoulders, sternoclavicular joints, elbows, wrists, hip joints, greater trochanters, ischial tuberosities, and knee joints. FDG accumulation was also observed in the surgical wound on the right anterior chest after an aortic valve replacement. (B) ^18^F-FDG-PET/CT showed FDG uptake in lumbar spinous processes and hip joints.

The use of ^18^F-FDG-PET/CT is currently supported in the diagnostic process of fever of unknown origin ^(1), (2)^. Furthermore, this modality has emerged as a gold standard investigation for PMR ^(3)^. Its combined pathognomonic PET/CT findings include FDG uptake in the periarticular regions of the shoulders and hips, in the interspinous regions, and adjacent to the ischial tuberosities ^(3)^. PET/CT also helps to identify coexistent vasculitis and exclude relevant differential diagnoses including clinically silent extra septic foci ^(3), (4)^.

## Article Information

### Conflicts of Interest

None

### Author Contributions

HS acquired data and drafted the manuscript. HO and MO reviewed and supervised the manuscript.

### Informed Consent

We have obtained informed consent for this manuscript.
